# Epithelial Cells Activate Plasmacytoid Dendritic Cells Improving Their Anti-HIV Activity

**DOI:** 10.1371/journal.pone.0028709

**Published:** 2011-12-07

**Authors:** Christian Rodriguez Rodrigues, Mercedes Cabrini, Federico Remes Lenicov, Juan Sabatté, Ana Ceballos, Carolina Jancic, Silvina Raiden, Matías Ostrowski, Claudia Silberstein, Jorge Geffner

**Affiliations:** 1 Centro Nacional de Referencia para el SIDA, Facultad de Medicina, Universidad de Buenos Aires, Argentina; 2 IIHEMA, Academia Nacional de Medicina, Buenos Aires, Argentina; 3 Laboratorio de Fisiopatogenia, Departamento de Fisiología, Facultad de Medicina, Universidad de Buenos Aires, Buenos Aires, Argentina; University of Pittsburgh, United States of America

## Abstract

Plasmacytoid dendritic cells (pDCs) play a major role in anti-viral immunity by virtue of their ability to produce high amounts of type I interferons (IFNs) and a variety of inflammatory cytokines and chemokines in response to viral infections. Since recent studies have established that pDCs accumulate at the site of virus entry in the mucosa, here we analyzed whether epithelial cells were able to modulate the function of pDCs. We found that the epithelial cell lines HT-29 and Caco-2, as well as a primary culture of human renal tubular epithelial cells (HRTEC), induced the phenotypic maturation of pDCs stimulating the production of inflammatory cytokines. By contrast, epithelial cells did not induce any change in the phenotype of conventional or myeloid DCs (cDCs) while significantly stimulated the production of the anti-inflammatory cytokine IL-10. Activation of pDCs by epithelial cells was prevented by Bafilomycin A1, an inhibitor of endosomal acidification as well as by the addition of RNase to the culture medium, suggesting the participation of endosomal TLRs. Interestingly, the cross-talk between both cell populations was shown to be associated to an increased expression of TLR7 and TLR9 by pDCs and the production of LL37 by epithelial cells, an antimicrobial peptide able to bind and transport extracellular nucleic acids into the endosomal compartments. Interestingly, epithelium-activated pDCs impaired the establishment of a productive HIV infection in two susceptible target cells through the stimulation of the production of type I IFNs, highlighting the anti-viral efficiency of this novel activation pathway.

## Introduction

Plasmacytoid dendritic cells (pDCs) play a critical role in anti-viral immunity. These cells develop fully in the bone marrow and are released into the blood stream comprising about 0.2% to 0.5% of peripheral blood mononuclear cells [1]–[3]. Recognition of viral nucleic acids by TLR7 and TLR9 triggers the activation of pDCs. This results in an increased expression of costimulatory and MHC class I and class II molecules, the production of inflammatory cytokines and specially the production of large amounts of type I IFNs, almost 100 to 1000-fold higher than the production mediated by other cell types [4], [5]. Not only viral nucleic acids but also host DNA appears to be able to activate pDCs. Studies performed in LES and psoriasis models suggest that recognition of self DNA by TLR9 triggers a sustained production of type I IFNs which promotes T cell-mediated autoimmunity favoring disease progression [4], [6].

Under steady-state conditions pDCs migrate from the peripheral blood to the T-cell rich areas of lymph nodes, mucosal-associated lymphoid tissues and spleen [7], [8]. Human blood pDCs express L-selectin and PSGL1, the counter-ligand of P- and E- selectins. They drive the emigration of pDCs from the blood into lymph nodes across high endothelial venules [7], [8]. pDCs are usually difficult to detect in peripheral tissues such as skin and mucosa. However, high numbers of pDCs have been found in injured tissues of autoimmune patients with lupus erythematosus (LES), psoriasis, Sjogren's syndrome, and multiple sclerosis [4], [9]. Moreover, during the course of viral infections large numbers of pDCs are recruited to inflamed mucosa providing innate immune protection against mucosal viral infection in situ [3], [4], [9]–[12]. These observations suggest that under different pathologic conditions pDCs are recruited to the mucosa in the proximity of epithelial cells that line mucosal surfaces.

The infiltration of pDCs into infected or inflamed tissues appears to involve the participation of a number of chemokine receptors such as CCR1, CCR2, CCR5, CXCR3 and CXCR4 [7], [8]. pDCs also express CCR9, the receptor for the chemokine CCL25, which drives the homing of pDCs to the small intestine [7], [8]. Not only chemokines, but also compounds released or produced in the context of tissue damage, such as adenosine, the heme-binding protein fragment peptide F2L, and C5a appear to participate in the recruitment of pDCs to inflamed tissues by interacting with the specific receptors A1, the formyl peptide receptor known as FPRL2, and the C5a receptor, respectively [13]–[15]. Finally, pDCs express ChemR23, a G-protein-coupled receptor, which drives the migration of pDCs in response to chemerin, a chemoattractant released by inflamed tissues and tumors [16].

Most viral infections are transmitted through mucosal epithelium, which provides the first line of defense against invading pathogens. The fact that pDCs accumulate at site of virus entry in the mucosa open the question whether epithelial cells were able to modulate the function of pDCs. A large number of studies have analyzed the ability of epithelium to modulate the function profile of conventional or myeloid dendritic cells (cDCs). By contrast, to our knowledge, no previous studies have analyzed the influence of epithelium on the function of pDCs. In this study we show that epithelial cells induce the activation of pDC. Epithelial cells efficiently stimulated the phenotypic maturation of pDCs, the production of inflammatory cytokines and improved the anti-HIV activity of pDCs. Our results support a new mechanism through which epithelial cells might contribute to host protection against virus infection.

## Results

### Epithelial cells induce the phenotypic maturation and the production of inflammatory cytokines by pDCs

**Figure 1:**
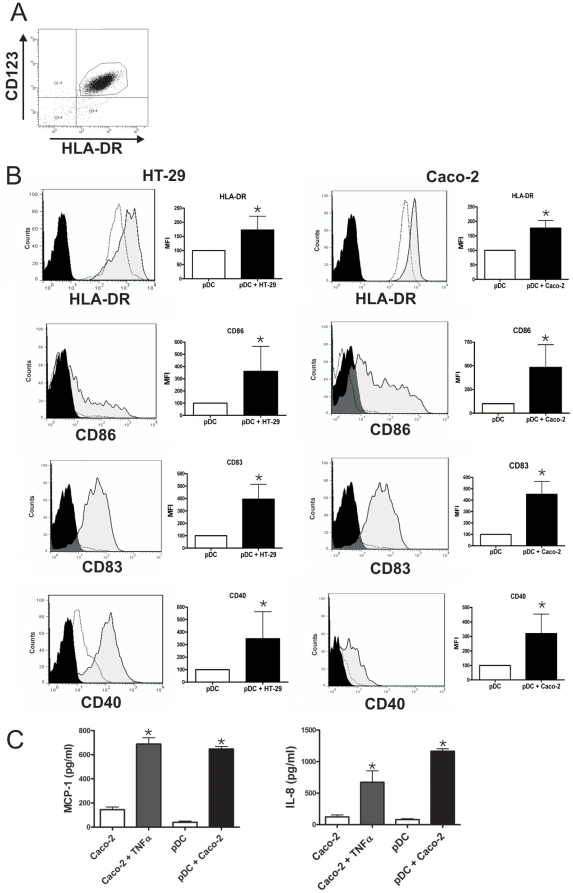
Epithelial cells induce the phenotypic maturation of pDCs. The epithelial cell lines HT-29 and Caco-2 were grown to confluence in 96 well flat bottom plates. pDCs (1×10^5^/200 µl) were cultured alone (controls) or with epithelial cells for 12 h. Then, pDCs were harvested and their phenotype was analyzed by flow cytometry. (**A**) Dot-plots illustrating the purity of pDCs and the expression of CD123 and HLA-DR. (**B**) The expression of HLA-DR, CD86, CD83, and CD40 in the gate of CD123+ cells is shown for pDCs cultured alone (open histograms) or in the presence of epithelial cells (grey-filled histograms). Black-filled histograms represent isotype controls (they were similar for pDCs cultured alone or in the presence of epithelial cells). A representative experiment (n = 5–7) is shown. Graph bars show the relative mean fluorescence intensity (MFI) of HLA-DR, CD86, CD83, and CD40 in the gate of CD123+ cells, for pDCs cultured alone or in the presence of epithelial cells. The MFI of pDCs cultured alone is assigned the value of 100. Results are the mean ± SEM of 5–7 experiments. (* p<0.05 vs pDCs cultured alone). (**C**) Caco-2 cells were grown to confluence in 96 well flat bottom plates. Cells were then cultured for 12 h in the absence or presence of TNF-α (50 ng/ml) or pDCs (1×10^5^/200 µl) and the production of the chemokines MCP-1 and IL-8 was assessed by ELISA. Results are the mean ± SEM of four experiments performed in duplicate. (*p<0.05 vs controls).

pDCs were purified from the blood of healthy adult human volunteers. PBMCs were isolated using Ficoll-Hypaque density centrifugation, and pDCs were positively selected using BDCA-4 magnetic beads. The purity of pDCs was higher than 93% (range 93–98%) and their expression of CD123 and HLA-DR is shown in **Figure 1A**. In a first set of experiments we analyzed whether two epithelial cell lines, HT-29 and Caco-2 were able to up-regulate the expression of HLA-DR, CD86, CD83, and CD40 in pDCs. Cell lines were grown to confluence in 96 well, flat bottom plates, and 1×10^5^ pDCs were added to each well in a final volume of 0.2 ml. Control pDCs were cultured alone. After 12 h of culture, pDCs were harvested from the culture and their phenotype was analyzed by flow cytometry. **Figure 1B** shows that both, HT-29 and Caco-2 cell lines effectively induced the up-regulation of HLA-DR, CD86, CD83 and CD40 in pDCs while the expression of CCR7 remained unchanged (not shown). By contrast, no stimulatory effect was observed when pDCs were cultured with non-epithelial cells such as the T cell line MT-2, the osteosarcoma cell line GHOST, or human fibroblasts (data not shown).

Further studies were done to analyze the degree of activation of the epithelial cell lines used in our experiments. It was performed by studying the production of the chemokines MCP-1 and IL-8. Previous studies have shown that both chemokines are produced at very low levels in resting Caco-2 cells and their production is strongly stimulated upon cellular activation [17], [18]. **Figure 1C** shows that Caco-2 cells cultured alone produce very low amounts of IL-8 and MCP-1 suggesting that they are in a resting state. As expected, treatment with TNF-α resulted in a marked stimulation in the production of both chemokines. Interestingly, the culture of Caco-2 cells with pDCs also resulted in a marked stimulation of the production of MCP-1 and IL-8 (**Figure 1C**), suggesting that the culture of pDCs with epithelial cells results, not only in the phenotypic maturation of pDCs, but also in the activation of epithelial cells.

**Figure 2:**
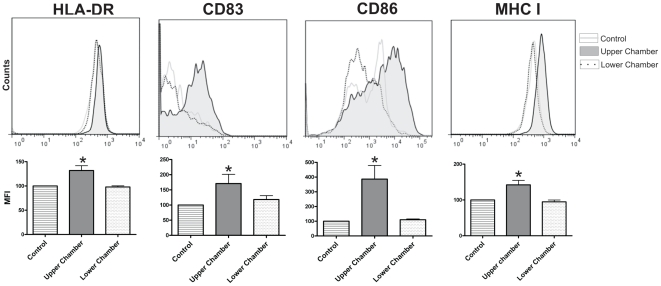
Epithelial cells induce the phenotypic maturation of pDCs in a cell contact-dependent manner. Experiments were performed using 24-transwell chambers with a polycarbonate filter (0.2 µm pore size). HT-29 cells were grown to confluence on the filter. pDCs (3×10^5^) were cultured alone in the lower chamber or in contact with the monolayer of epithelial cells, in the upper chamber. Control cells were cultured in the upper chamber without epithelial cells. After 12 h of culture, pDCs were harvested and the expression of HLA-DR, CD83, CD86, and MHC class I was analyzed in the gate of CD123^+^ cells by flow cytometry. The relative mean fluorescence intensity (MFI) of isotype controls were in all cases lower than 5 (not shown). The MFI for control cells (for all the markers analyzed) was assigned to the value of 100, and the MFI for pDCs cultured alone in the lower chamber (X) or those cultured in contact with the monolayer of epithelial cells in the upper chamber (Y) was calculated using the equation: X or Y×100/MFI of control pDCs. Histograms show a representative experiment (n = 4–8). Graph bars show the MFI of HLA-DR, CD83, CD86, and MHC class I in the gate of CD123^+^ cells. Results are the mean ± SEM of 6–7 experiments. (* p<0.05 vs control).

To analyze whether activation of pDCs by epithelial cells required the physical interaction between both cell populations, a new set of experiments was performed using 24-transwell chambers with a polycarbonate filter (0.2 µm pore size). HT-29 cells were grown to confluence on the filter, and pDCs were cultured alone in the lower chamber or together with epithelial cells in the upper chamber. Cells were cultured for 12 h at 37°C and the expression of HLA-DR, CD83, CD86 and class I HLA molecules was then analyzed by flow cytometry. **Figure 2** shows that pDCs cultured together with epithelial cells in the upper chamber up-regulated the expression of all the markers analyzed. By contrast, no changes in the phenotype of pDCs were observed for those cells incubated alone in the lower chamber. This suggests that epithelial cells activate pDCs in a cell contact-dependent manner. Consistent with this notion, we observed that pDCs did not increase the expression of HLA-DR, CD83, CD86 and class I HLA molecules when cultured for 12 h with supernatants collected from confluent Caco-2 or HT-29 cells (not shown).

**Figure 3:**
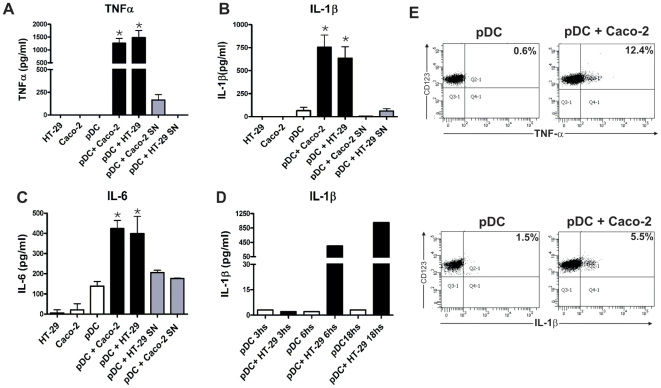
Epithelial cells stimulate the production of inflammatory cytokines by pDCs. (**A–C**) The epithelial cell lines HT-29 and Caco-2 were grown to confluence in 96 well flat bottom plates. pDCs (1×10^5^/200 µl) were cultured alone (controls), with HT-29 or Caco-2 cells, or with supernatants (SN) collected from confluent HT-29 or Caco-2 cells cultured for 12 h. Then, the production of TNF-α, IL-1β, and IL-6 was evaluated in cell supernatants by ELISA. Results are the mean ± SEM of eight experiments performed in duplicate. (*p<0.05 vs controls). The levels of TNF-α and IL-1β produce by HT-29 and Caco-2 cells cultured alone were undetectable (below the detection limit of the ELISA assays). (D) Kinetic of the production of IL-1β by pDCs cultured with the epithelial cell line HT-29. A representative experiment (n = 2) is shown. (E) Caco-2 cells were grown to confluence in 96 well flat bottom plates. pDCs (1×10^5^/200 µl) were cultured alone or with Caco-2 cells for 12 h. In the assays directed to evaluate the production of TNF-α, Brefeldin A (10 µg/ml) was added during the last 6 h of culture. Then, the production of TNF-α and IL-1β was evaluated by intracellular staining and flow cytometry. A representative experiment (n = 3) is shown.

We next analyzed whether epithelial cells were also able to stimulate the production of inflammatory cytokines by pDCs. To this aim, epithelial cells and pDCs were cultured together for 12 h and the levels of TNF-α, IL-1β, and IL-6 were measured in cell supernatants by ELISA. **Figures 3 A–C** show that very low or undetectable levels of these cytokines were detected when epithelial cells or pDCs were cultured alone. By contrast, high levels of TNF-α, IL-1β, and IL-6 were observed when epithelial cells and pDCs were cultured together. Supernatants collected from Caco-2 or HT-29 cells grown to confluence failed to induce any production of cytokines by pDCs suggesting that epithelial cells stimulate the production of inflammatory cytokines by pDCs in a cell contact-dependent manner. The kinetic of IL-1β production is shown in **Figure 3D**. High levels of IL-1β production were observed as early as 6 h after the addition of pDCs to confluent epithelial cells. As expected, by analyzing the presence of intracellular TNF-α and IL-1β by flow cytometry we found that pDCs (**Figure 3E**), but not epithelial cells (data not shown), were the source of these cytokines.

**Figure 4:**
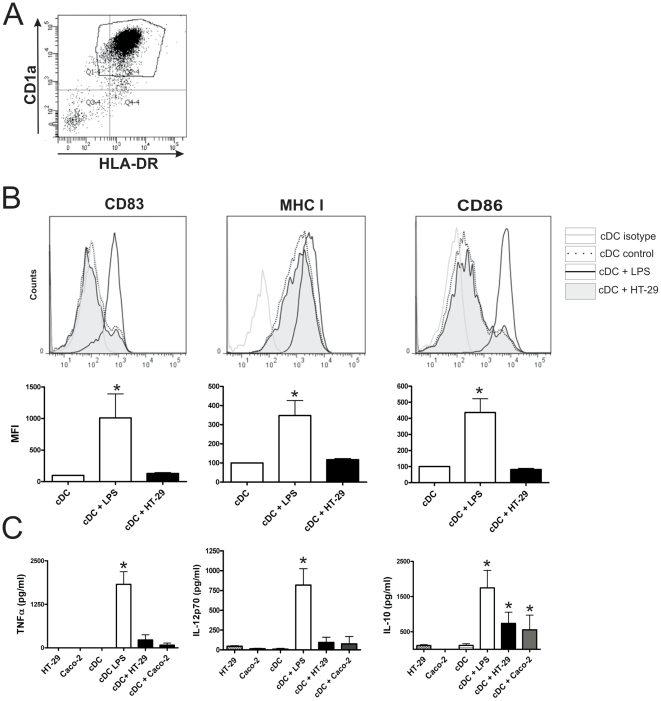
Epithelial cells induce neither the phenotypic maturation nor the stimulation of the production of inflammatory cytokines by cDCs. Conventional DCs were obtained from human monocytes (>85% purity) cultured for 5 days with GM-CSF plus IL-4. The epithelial cell lines HT-29 and Caco-2 were grown to confluence in 96 well, flat bottom plates. DCs (1×10^5^/200 µl) were cultured alone for 12 h, in the absence or presence of 100 ng/ml of LPS or in the presence of confluent monolayers of HT-29 or Caco-2 cells, in 96 well flat bottom plates. (**A**) Dot-plots illustrating the purity of cDCs and the expression of CD1a and HLA-DR. (**B**) The expression of CD83, MHC class I, and CD86 was analyzed by flow cytometry in the gate of CD1a+ cells and a representative experiment (n = 5–7) is shown. Graph bars show the MFI of CD83, MHC class I, and CD86. The MFI of cDCs cultured alone is assigned the value of 100. Results are the mean ± SEM of 7–10 experiments. (* p<0.05 vs cDCs). (**C**) The production of TNF-α, IL-12p70, and IL-10 was evaluated in cell supernatants by ELISA. Results are the mean ± SEM of 7–10 experiments performed in duplicate. (* p<0.05 vs controls).

We then asked whether epithelial cells were also able to activate conventional or myeloid DCs (cDCs). These cells were obtained from human monocytes cultured for 5 days with GM-CSF plus IL-4. As described for pDCs, cDCs (1×10^5^ cells, purity >85%) were cultured for 12 h with confluent monolayers of HT-29 cells in 96 well, flat bottom plates, in a final volume of 0.2 ml. Then, cDCs were harvested and their phenotype was analyzed by flow cytometry. As a positive control for the induction of phenotypic maturation of cDCs we used LPS-treated cDCs. Contrasting with the results observed for pDCs, HT-29 cells did not induce any change in the phenotype of cDCs (**Figures 4A and B**). Moreover, as shown in **Figure 4C**, HT-29 and Caco-2 cells were unable to stimulate the production of the inflammatory cytokines TNF-α and IL-12p70 while they significantly stimulated the production of IL-10. We conclude that pDCs, but not cDCs, are activated in a pro-inflammatory profile by epithelial cells.

### Primary human renal tubular epithelial cells (HRTEC) induce the phenotypic maturation and the production of inflammatory cytokines by pDCs

**Figure 5:**
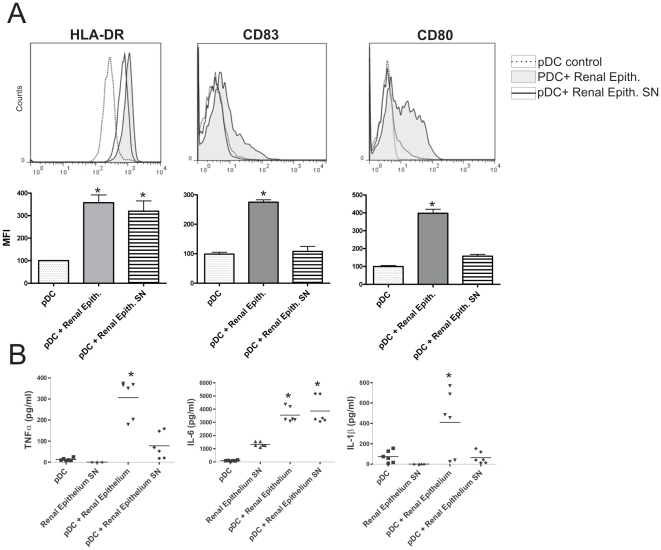
Primary human renal tubular epithelial cells (HRTEC) induce the activation of pDCs. Primary human renal proximal tubular cells, obtained as described under Materials and Methods, were grown to confluence in 96 well, flat bottom plates. pDCs (1×10^5^/well) were cultured for 12 h alone (controls), with confluent HRTEC, or with supernatants collected from confluent HRTEC incubated alone for 12 h. Then, the phenotype of pDCs in the gate of CD123+ cells was analyzed by flow (A) Histograms of representative experiments (n = 6) are shown. The MFI of isotype controls were in all cases lower than 5 (not shown). Graph bars show the relative mean fluorescence intensity (MFI) of HLA-DR, CD83, and CD80 for pDCs cultured alone or in the presence of epithelial cells or epithelial cell supernatants. The MFI of pDCs cultured alone is assigned the value of 100. Results are the mean ± SEM of 7–8 experiments performed in duplicate. (* p<0.05 vs pDCs). (B) The production of TNF-α, IL-6, and IL-1β were assessed in cell supernatants by ELISA. *p<0.05 vs pDCs.

Our previous results were obtained using epithelial cell lines. In order to establish whether primary cultures of epithelial cells were also able to activate pDCs, we performed a new set of experiments using primary human renal proximal tubular cells, obtained as described under Materials and Methods. Cells were grown to confluence in 96 well, flat bottom plates. pDCs were added (1×10^5^/well) to confluent epithelial cells and after 12 h of culture the phenotype of pDCs was analyzed by flow cytometry. **Figure 5A** shows that HRTEC increase the expression of HLA-DR, CD83, and CD80 by pDCs in a similar fashion than the epithelial cell lines HT-29 and Caco-2. Moreover, HRTEC markedly increased the production of TNF-α, IL-6, and IL-1β by pDCs (**Figure 5B**). Interestingly, and in contrast with the observations made with the epithelial cell lines HT-29 and Caco-2 we found that supernatants from HRTEC cultured alone significantly induced the up-regulation of HLA-DR expression and the stimulation of IL-6 production by pDCs, suggesting that primary epithelial cells might activate pDCs, at least in part, through cell contact-independent mechanisms.

### Analysis of the mechanisms through which epithelial cells induce the activation of pDCs

**Figure 6:**
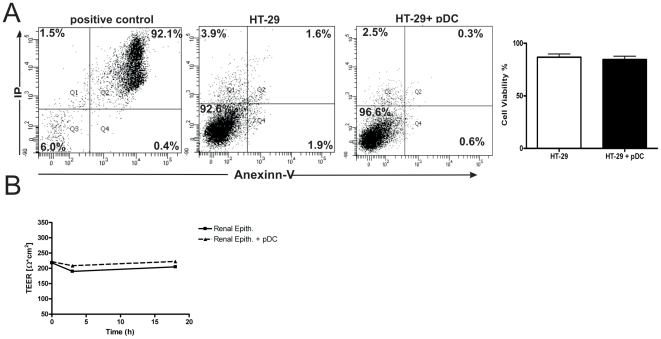
Plasmacytoid DCs do not affect the integrity of epithelial cells. (**A**) The epithelial cell line HT-29 was grown to confluence in 96 well flat bottom plates. Then, they were cultured in the absence or presence of pDCs (1×10^5^/200 µl) for 18 h, and the viability of epithelial cells was analyzed by flow cytometry using Annexin V and propidium iodide. Representative dot-plots are shown. Positive control represents epithelial cells culture for 18 hs in culture medium without fetal calf serum (protein-free medium). Graph bars show the percentages of epithelial cell viability for epithelial cells cultured alone or in the presence of pDCs. Results are the mean ± SEM of 4 experiments performed in duplicate. (**B**) Primary human renal tubular epithelial cells (HRTEC) were grown to confluence on a polycarbonate filter (0.2 µm pore size) in the upper chamber of a 24-transwell plate. Then, epithelial cells were incubated for 18 h together with pDCs (2×10^5^) in the upper chamber or in the absence of pDCs. At different time points the transepithelial electrical resistance (TEER) was measured, as described under Materials and Methods. A representative experiment (n = 3) is shown.

Plasmacytoid DCs might induce deleterious effects on epithelial cells leading to the expression or release of damage associated molecular patters (DAMPs) or self-nucleic acid which in turn induce the activation of pDCs. In a first set of experiments we analyzed whether pDCs might affect epithelial cell integrity. The cell line HT-29 was incubated for 18 h with or without pDCs and the viability of epithelial cells was then analyzed by flow cytometry using Annexin V and propidium iodide. **Figure 6A** shows that pDCs did not induce deleterious effects on epithelial cell integrity. Positive control represents epithelial cells cultured for 18 h in protein-free medium (cell death >90%). Further studies were then performed by analyzing the transepithelial electrical resistance (TEER) of HRTEC cultured with or without pDCs. HRTEC were grown to confluence on a polycarbonate filter (0.2 µm pore size) in the upper chamber of a 24-transwell plate. Then, epithelial cells were incubated for 18 h together with pDCs (2×10^5^) in the upper chamber or without pDCs. At different time points TEER was measured, as described under Materials and Methods. As shown in **Figure 6B** the culture of HRTEC with pDCs did not result in any change in the transepithelial resistance of HRTEC. We conclude that pDCs do not affect epithelial cell integrity.

**Figure 7:**
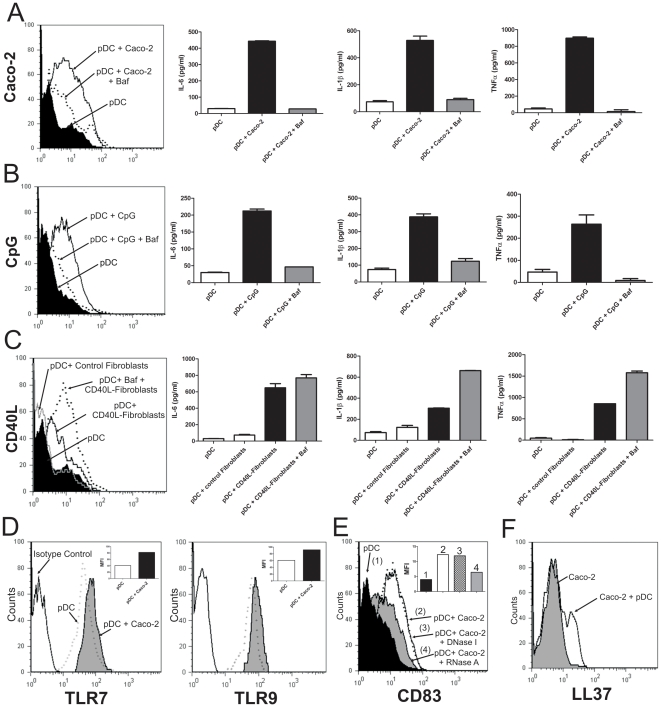
Analysis of the mechanisms through which epithelial cells induce the activation of pDCs. (**A–C**) pDCs (1×10^5^/200 µl) were pre-incubated or not with Bafilomycin A1 (50 ng/ml) for 30 min. Then, they were cultured for 12 h at 37°C alone or in the presence of confluent Caco-2 cells (**A**), the CpG-containing oliguncleotide 2006 (2.5 µg/ml) (**B**), confluent control fibroblasts or confluent CD40L-expressing fibroblasts (**C**). Then, the expression of CD83 and the production of the cytokines IL-6, IL-1β and TNF-α was evaluated by flow cytometry and ELISA, respectively. Representative histograms (n = 3–4) are shown. Graph bars show a representative experiment made by duplicate (**D**) pDCs (1×10^5^/200 µl) were cultured alone or in presence of confluent Caco-2 cells for 12 h. Then, the expression of TLR7 and TLR9 was evaluated by intracellular staining and flow cytometry. A representative experiment (n = 3) is shown. (**E**) Caco-2 were grown to confluence in 96 well flat bottom plates. pDCs (1×10^5^/200 µl) were cultured alone (controls) or with Caco-2 cells for 12 h, in the absence or presence of DNase I (15 µg/ml) or RNase A (10 µg/ml). Then, pDCs were harvested and the expression of CD83 was analyzed by flow cytometry in the gate of CD123-positive cells. Dot-plots from a representative experiment (n = 4) are shown. (**F**) Caco-2 cells were grown to confluence in 96 well flat bottom plates. They were cultured for 12 h with or without pDCs (1×10^5^/200 µl). Then, the expression of LL37 by Caco-2 cells was analyzed by intracellular staining and flow cytometry, in the gate of CD123-negative cells. A representative experiment (n = 4) is shown.

The main pathway leading to the activation of pDCs is mediated by the recognition of RNA and DNA by TLR7 and TLR9, respectively [1], [5]. Not only microbial nucleic acids, but also self-nucleic acids are able to activate endosomal TLRs [4], [6]. Even though pDCs did not induce deleterious effects on epithelial cells, we speculated that the cross-talk between epithelial cells and pDCs could sensitize pDCs to the activation by self-nucleic acids, which might gain access to the extracellular space through two major mechanisms; the spontaneous death of a reduced fraction of epithelial cells or the active release by epithelial cells of small membrane vesicles containing nucleic acids (exosomes) [19], [20]. To analyze the participation of endosomal TLRs in the activation of pDCs, and considering that the effective recognition of nucleic acids by endosomal TLRs requires the maturation and acidification of the endosomes [21], we carried out a new set of experiments using Bafilomycin A1, an antagonist of the vacuolar type proton ATPase responsible for endosomal acidification [22]. **Figure 7A** shows that Bafilomycin A1 markedly prevented the up-regulation of CD83 in pDCs as well as the production of IL-6, IL-1β, and TNF-α stimulated by Caco-2 cells. As expected, Bafilomicyn A1 almost completely abrogated the activation of pDCs by CpG-containing oligonucleotides (**Figure 7B**) without affecting the activation of pDCs induced by CD40L-expressing fibroblasts (**Figure 7C**). Together, these results suggest that endosomal TLRs are involved in the activation of pDCs induced by epithelial cells.

Interestingly, when exploring the expression of TLR7 and TLR9 in pDCs we found that the expression of both receptors was up-regulated as a consequence of the incubation of pDCs with epithelial cells (**Figure 7C**). This suggests that epithelial cells could sensitize pDCs to endosomal TLR ligands. Moreover, supporting that self-nucleic acids are involved in the activation of pDCs by epithelial cells, we observed that depletion of extracellular RNA from the cultures by RNase A prevented the up-regulation of CD83 expression in pDCs induced by epithelial cells. By contrast, no inhibitory effect was observed using DNase I (**Figure 7D**). These results suggest that self-RNA, but not self-DNA, is involved in the activation of pDCs. Usually, self-nucleic acids are rapidly degraded in the extracellular space and fail to access to endosomal TLRs [23]. This explains why the culture with necrotic cells does not lead to the activation of pDCs [24]–[26]. In line with this, we did not observe any activation of pDCs when they were cultured with necrotic (freeze-thawed) Caco-2 cells (data not shown). Recently, Ganguly and colleagues [27] showed that the antimicrobial peptide LL37 binds self-RNA and promotes RNA transport into the endosomal compartments of pDCs enabling extracellular RNA to activate TLR7. Since activated epithelial cells are one of the major sources of LL37 [28], we evaluated whether incubation with pDCs resulted in an increased expression of LL37 by Caco-2 cells. Results in **Figure 7E** show that pDCs enhance the expression of LL37 in Caco-2 cells. Overall, these results suggest that the cross-talk between pDCs and epithelial cells leads to the activation of both cell populations enhancing the ability of pDCs to sense extracellular self-RNA.

### Epithelial cells improve the anti-HIV activity mediated by pDCs

**Figure 8:**
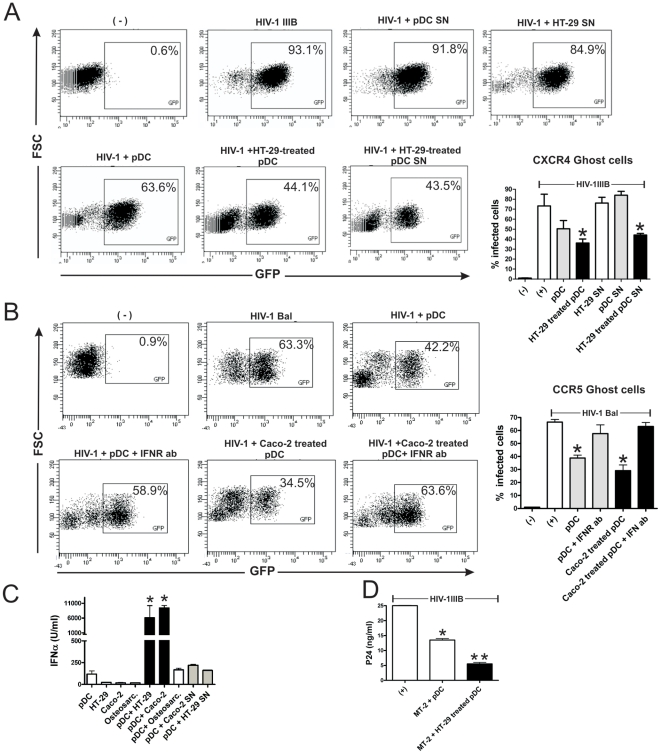
Epithelial cells improve the anti-HIV activity mediated by pDCs. (**A**) GHOST cells expressing CD4, CXCR4, and a Tat-dependent green fluorescent protein reporter cassette were cultured in 24-well flat bottom plates (5×10^4^ cells/well) with or without HIV-1_IIIB_ (50 ng p24) for 2 h at 37°C. Cells were then washed and incubated with 1×10^5^ pDCs (untreated or preincubated with HT-29 cells for 12 h) or supernatants collected after 12 h of culture of pDCs, HT-29 confluent cells, or pDCs cultured together with HT-29 cells. After 48 h, the infection of GHOST cells was analyzed by flow cytometry by studying the percentage of GFP+ cells. A representative experiment (n = 4–5) is shown. Graph bars show the percentage of GHOST cells infected by HIV. Results are the mean ± SEM of 4–5 experiments performed in duplicate. *p<0.05 vs GHOST cells incubated only with HIV-1IIIB (+). (**B**) GHOST cells expressing CD4, CCR5, and a Tat-dependent green fluorescent protein reporter cassette were cultured in 24-well flat bottom plates (5×10^4^ cells/well) with or without HIV-1 BaL (50 ng p24) for 2 h at 37°C. Cells were then washed and incubated with 1×10^5^ pDCs (untreated or preincubated with Caco-2 cells for 12 h). When indicated, a blocking antibody directed to type I IFN receptor (50 µg/ml) was added to the cultures before the addition of pDCs. After 48 h, the infection of GHOST cells was analyzed by flow cytometry by studying the percentage of GFP+ cells. A representative experiment (n = 4) is shown. Graph bars show the percentage of GHOST cells infected by HIV-1. Results are the mean ± SEM of 4 experiments performed in duplicate. (* p<0.05 vs GHOST cells incubated only with HIV-1 BaL). (**C**) The epithelial cell lines HT-29 and Caco-2, and the osteosarcoma line GHOST (osteosarc) were grown to confluence in 96 well flat bottom plates. pDCs (1×10^5^/200 µl) were cultured for 12 h alone (controls), with HT-29, Caco-2 cells, GHOST cells or with supernatants collected from HT-29 or Caco-2 grown to confluence and cultured for 12 h. Then, the production of IFN-α was evaluated in cell supernatants by ELISA. Results are the mean ± SEM of five experiments performed in duplicate. (* p<0.05 vs controls). (D) The T cell line MT-2 (1×10^5^ cells) was cultured with HIV-1_IIIB_ (50 ng p24) for 2 h at 37°C. Cells were then washed and incubated with 1×10^5^ pDCs, previously incubated for 12 h with or without confluent HT-29 cells. Infection of MT-2 cells was determined by measuring p24 antigen levels in cell supernatants 48 h post-infection. Results are the mean ± SEM of four experiments performed in duplicate. (* p<0.05 vs MT-2+HIV-1 and ** p<0.05 vs MT-2+HIV-1+pDCs).

Previous studies have shown that pDCs prevent the infection of target cells by HIV in vitro [29], [30]. To analyze whether epithelial cells were able to improve the anti-HIV activity mediated by pDCs, we performed a new set of experiments using GHOST cells expressing CD4, the coreceptor CXCR4 and a Tat-dependent green fluorescent protein (GFP) reporter cassette. GHOST cells were cultured with HIV-1_IIIB_ (X4 tropic) in the absence or presence of pDCs previously incubated, or not, with confluent epithelial cells. Infection was detected by flow cytometric quantitation of GFP-expressing cells, after 48 h of culture. **Figure 8A** shows that pDCs prevented the infection of GHOST cells and that this anti-HIV activity was further improved using pDCs preincubated with epithelial cells during 12 h. No anti-HIV activity was observed using supernatants collected from pDCs or HT-29 cells cultured alone. By contrast, supernatants collected from pDCs cultured for 12 h with monolayers of HT-29 cells induced a strong anti-HIV activity. This supports that factors released by HT-29-treated pDCs are responsible for the anti-HIV activity. Similar results were observed when GHOST cells expressing CD4, the coreceptor CCR5 and a Tat-dependent green fluorescent protein (GFP) reporter cassette were infected with HIV-1 BaL (R5 tropic). **Figure 8B** shows that pDCs prevented GHOST cell infection by HIV-1 BaL, and that this anti-HIV activity was further improved using pDCs preincubated with epithelial cells. Since type I IFNs represent the most important anti-viral factors produced by pDCs we also analyzed whether they account for the anti-viral effect mediated by pDCs. **Figure 8B** shows that the addition of a blocking antibody directed to the receptor for type I IFNs almost completely prevented the anti-viral effect mediated by pDCs. Consistent with these results, **Figure 8C** shows that the epithelial cell lines HT-29 and Caco-2, but not the osteosarcoma cell line GHOST, markedly stimulated the production of IFN-α by pDCs. We finally analyzed the anti-HIV activity of pDCs using the T-cell line MT-2 as target cell. Infection was evaluated by measuring the levels of the HIV-1 antigen p24 in cell supernatants by ELISA. Consistent with the results observed using GHOST cells, we found that preincubation with HT-29 cells resulted in the stimulation of the anti-HIV activity mediated by pDCs (**Figure 8D**).

## Discussion

In the present study we show for the first time that epithelial cells activate pDCs. Activation of pDCs results in an increased expression of molecules responsible for antigen presentation, the production of inflammatory cytokines, and an improved ability of pDCs to prevent the infection of target cells by HIV through a pathway dependent on the production of type I IFNs. Previous reports from the Rescigno group [31]–[34] showed that through the release of soluble factors such as TGF-β, retinoic acid, and thymic stromal lymphopoietin (TSLP), epithelial cells efficiently stimulate the differentiation of cDCs into a tolerogenic profile able to drive the development of CD4^+^CD25^+^Foxp3^+^ regulatory T cells. Consistent with these observations we found that epithelial cells stimulated the production of IL-10 without inducing the phenotypic maturation or the production of inflammatory cytokines by cDCs. Together, these observations support the notion that epithelial cells exert opposite effects on pDCs and cDCs.

The main pathway leading to the activation of pDCs is mediated by the recognition of single-stranded RNA and double-stranded DNA by TLR7 and TLR9, respectively. This leads to the recruitment of MyD88 and to the assembly of a multiprotein signal transduction complex responsible for the activation of IRF7, the master transcription factor involved in the induction of type I IFNs that is expressed at high constitutive levels in pDCs but not other PBMC populations [4], [5]. It should be noted that, contrasting with cDCs which can be fully activated by a broad range of stimuli including a variety of pathogen-associated molecular patterns (PAMPs), danger-associated molecular patterns (DAMPs), cytokines and chemokines [35]–[37], only few stimuli besides microbial nucleic acids appear to be able to activate pDCs. Among them, CD40L and endothelial microparticles resulting from vascular endothelium injury. It has been shown that upon activation by CD40L, pDCs up-regulate antigen presenting, adhesion and costimulatory molecules increasing the production of type I IFNs and their ability to activate naive T cells [1], [38], [39]. Contrasting results, on the other hand, have been published regarding the ability of CD40L-stimulated pDCs to drive TCD4+ response into a Th1 vs Th2 polarization profile [1], [38], [39]. A different pattern of activation is induced by endothelial microparticles which stimulated the up-regulation of costimulatory molecules, the production of the inflammatory cytokines IL-6 and IL-8 but were completely unable to stimulate the production of type I IFNs [40].

The ability to activate pDCs appears to be selectively expressed by epithelial cells. In fact, no activation of pDCs was observed when they were cultured with non-epithelial cells such as the T cell line MT-2, the osteosarcoma cell line GHOST, or human fibroblasts (Rodriguez Rodrigues C, unpublished results). While the early events involved in the cross-talk between pDCs and epithelial cells remain to be defined, our results suggest that incubation with epithelial cells sensitize pDCs to effectively recognize self-nucleic acids released in the extracellular space. In fact, we found that the activation of pDCs was markedly inhibited by the addition of RNase as well as by the inhibitor of endosomal maturation Bafilomycin A1, suggesting that the recognition of self-RNA by endosomal TLRs is involved. This recognition might be facilitated by changes in both cell populations; an enhanced expression of endosomal TLRs by pDCs, and the increased production of the antimicrobial peptide LL37 by epithelial cells. In line with these results, and consistent with previous reports [24]–[26], we observed that the exposure of pDCs to necrotic epithelial cells did not induce any activation of pDCs, suggesting that an active cross-talk between both cell populations is required.

Our observation indicating that epithelial cell lines activate pDCs in a contact-dependent mode could be explained considering that RNA is rapidly degraded in the extracellular space [23]. Moreover, the cationic nature of the antimicrobial peptide LL37 [27] might lead to its rapid association to cell membranes. How to explain the ability of primary epithelial cells cultures to activate pDCs, at least in part, through cell contact-independent mechanisms? Primary epithelial cells would display a higher ability than epithelial cell lines to release self-RNA, to produce the antimicrobial peptide LL37, and/or to stimulate the expression of endosomal TLRs by pDCs. Further experiments are needed to test these hypotheses.

Plasmacytoid DCs play a critical role in the immune response to different virus including HIV-1. Groot and colleagues [30] have shown that cDCs and pDCs have opposing roles on HIV-1 infection of T cells. The authors reported that cDCs enhance HIV-1 infection through the capture of the virus and subsequent transmission to T cells, and also that differently maturated cDCs have different HIV-1 transmission efficiencies. By contrast, pDCs inhibit HIV-1 replication in T cells through a mechanism dependent on the production of IFN-α and undefined small anti-viral molecules. Our results are in line with those of Groot et al. [30] and support that pDCs effectively prevent HIV-1 infection of target cells. However, the authors observed that unstimulated pDCs or pDCs activated by different maturation stimuli such as the TLR synthetic agonist R-848, poly (I:C) or fixed *Staphylococus aureus* Cowan strain I bacteria (SAC) show a similar ability to prevent the infection of T cells by HIV [30]. By contrast, our results show that activation of pDCs by epithelial cells results in an enhanced ability to inhibit HIV replication through a type I IFN-dependent pathway. Consistent with these results, Meyers and colleagues [29] have previously shown that activation by CpG ODN markedly increase the anti-HIV activity mediated by pDCs.

In conclusion, we have shown that epithelial cells are able to activate pDCs. Because pDCs are usually difficult to detect in the normal mucosa [7], [8], they would rarely interact with the epithelium under steady-state conditions. However, during the course of viral infections, autoimmune and allergic diseases, pDCs are recruited to the mucosa [13], [15], [41]–[43] thus favoring their interaction with epithelial cells lining the gastrointestinal, genitourinary, and respiratory tracts. This might enable the cross-talk between both cell populations leading to the activation of pDCs. This novel pathway of pDC activation may contribute not only to anti-viral immune response but also to the development of tissue injury in allergic and autoimmune processes.

## Materials and Methods

### Reagents

Lipopolysaccharide from *Escherichia coli*, trypsin, collagenase type I, dimethyl sulfoxide, endothelial cell growth factor, and recombinant human granulocyte-macrophage colony-stimulating factor (GM-CSF) were from Sigma-Aldrich (St. Louis, MO). Bafilomycin A1 (*Streptomyces griseus*) was from Merk (Darmstadt, Germany), RNase A (bovine pancreas) was from Fermentas (Buenos Aires, Argentine), DNase I (bovine pancreas) was from Invitrogen (Carlsbad, CA). Recombinant human interleukin-4 (IL-4) was from Preprotech (Rocky Hill, NJ) or R&D Systems (Minneapolis, MN). Ficoll-Hypaque and Percoll were from Amersham Pharmacia Biotech (Piscataway, NJ). The sequence of the phosphodiester CpG-containing oligonucleotide used was: 2006, TCGTCGTTTTGTCGTTTTGTCGTT (Sigma Genosys, St. Louis, MO).

### Isolation of pDCs and preparation of conventional dendritic cells (cDCs)

Peripheral blood mononuclear cells (PBMCs) were isolated from healthy volunteers by standard density gradient centrifugation on Ficoll-Hypaque. pDCs were isolated using immunomagnetic cell sorting (BDCA-4 cell isolation kit, Miltenyi Biotec; Germany) according to the manufacturer's instructions. The purity of pDCs was checked by FACS using MAb directed to CD123 and HLA-DR and was found to be >93%. Monocytes were purified by centrifugation on a discontinuous Percoll gradient with modifications of a previously described method [44]. Briefly, PBMCs were suspended in Ca^2+^/Mg^2+^-free Tyrode solution supplemented with 0.2% EDTA and incubated during 30 min at 37°C. During this incubation, the osmolarity of the medium was gradually increased from 290 to 360 osmol/liter by the addition of 9% NaCl. Three different Percoll fractions were layered in polypropylene tubes: 50% at the bottom, followed by 46 and 40%. PBMCs (5×10^6^/ml) were layered at the top, and they were centrifuged at 400× *g* for 20 min at 4°C. Monocytes were recovered at the 50/46% interface. The purity was checked by FACS using an anti-CD14 MAb and was found to be >85%. To obtain cDCs, monocytes were cultured in RPMI 1640 medium (Life Technologies, Grand Island, NY) supplemented with 10% heat-inactivated fetal calf serum, 50 U of penicillin/ml, 50 µg of streptomycin/ml (Life Technologies) (complete culture medium) at 10^6^ cells/ml with 10 ng/ml of IL-4 and 10 ng/ml of GM-CSF, as described by Sallusto and Lanzavecchia [45]. On day 5, the cells were analyzed by FACS.

### Cell lines and virus

Human intestinal HT-29 and Caco-2 cell lines (generous gift of Dr Martín Rumbo, National University of La Plata, Argentina) were grown at 37°C in Dulbecco's modified Eagle's medium (Cellgro-Mediatech, VA) containing 10% heat-inactivated fetal calf serum (Gibco; Invitrogen), penicillin/streptomycin (100 U/ml), 2 mM L-glutamin, 1 mM pyruvate, and 0.1 mM nonessential amino acids (all from Life Technologies, Grand Island, NY) at pH 7.3. Epithelial cells were removed from the wells using a solution of 0.25% Trypsin-EDTA 1 mM prepared in Hank's balanced salt solution without Ca^2+^ and Mg^2+^. The human osteosarcoma cell line GHOST, expressing CD4, the HIV coreceptors CXCR4 or CCR5, and a Tat-dependent green fluorescent protein reporter cassette [46], and the T cell line MT-2 were obtained through the AIDS Research and Reference Reagent Program, Division of AIDS, National Institute of Allergy and Infectious Disease, National Institutes of Health. GHOST cells were grown in DMEM medium (Cellgro-Mediatech) supplemented with 10% heat-inactivated fetal calf serum, L-glutamine (2 mM), penicillin/streptomycin (100 U/ml), G418 (300 µg/ml), hygromycin (100 µg/ml) and puromycin (1 µg/ml) (all from Life Technologies) in 24-well flat bottom plates (5×10^4^ cells/well). Cells were detached using 0.25% trypsin. Murine fibroblasts stably transfected with a human CD40L cDNA and their control counterpart were provided by Dr. Claire Hivroz from Institut Curie, Paris. The CXCR4-using HIV-1 IIIB and the CCR5-using HIV-1 BaL were from the AIDS Research and Reference Reagent Program. The HIV-1 IIIB isolate was obtained from H9HTLV-IIIB supernatants while HIV-1 BaL was grown in human monocyte-derived macrophages. The viruses were concentrated by ultracentrifugation at 28,000 rpm for 90 min at 4°C (L2-65B ultracentrifuge; Beckman Coulter), and the virus pellet was suspended in RPMI 1640 medium. p24 antigen levels were determined by ELISA (Vironostika, Biomerieux, Argentina), and virus input into assays was a function of p24 antigen concentration. The virus stocks as well as all the cell cultures were free of Mycoplasma as measured by the Mycoplasma PCR detection kit (Venor®GeM), Cambridge, UK).

### Isolation and culture of human renal tubular epithelial cells

Human renal tubular epithelial cells (HRTEC) were isolated from kidneys removed from different adult patients undergoing nephrectomies for renal cell carcinoma from the “Unidad de Urología, Hospital Nacional Profesor A. Posadas, Buenos Aires, Argentina”. The removal of the portion of the renal tissue for research purposes was approved by the ethics committee of the University of Buenos Aires. The cortex was dissected from the renal medulla and the primary culture of the HRTEC was performed as previously described [47]. Briefly, the cortical fragments were cultured for 1 h at 37°C in a buffer containing 0.1% collagenase type I. Cells were washed and suspended in RPMI 1640 medium supplemented with 5% heat-inactivated fetal calf serum, 2 mM L-glutamine, and 100 U/ml penicillin/streptomycin. Cells were grown at 37°C to confluence. The cell isolates were trypsinized, concentrated in heat-inactivated fetal calf serum containing 5% dimethyl sulfoxide and stored in liquid nitrogen for subsequent use. Cells were then cultured in flasks or in 96-well, flat bottom plates in complete culture medium supplemented with 1% endothelial cell growth factor and were used between three and five passages. By light microscopy, more than 95% of the cells had similar morphologies. These cells were confirmed as epithelial cells by positive staining for cytokeratins (Sigma-Aldrich). The presence of fibroblasts was ruled out by the lack of reactivity with an antibody directed to the human fibroblast common antigen (Dako, Denmark). Furthermore, the cells were also negative for staining with an antibody directed to the endothelial cell antigen PECAM (CD31) (Dako).

### Culture of pDCs with epithelial cells

Epithelial cells were grown to confluence in 96 well, flat bottom plates. When the cultures reached 90–100% confluence, 1×10^5^ pDCs were added to each well, and cells were cultured in 0.2 ml of RPMI 1640 culture medium supplemented with 10% heat-inactivated fetal calf serum, 50 U of penicillin/ml, 50 µg of streptomycin/ml. Plasmacytoid dendritic cells cultured without epithelial cells and epithelial cells cultured alone were used as controls. After 12 h of culture, the levels of the cytokines TNF-α, IL-1β, IL-6, IFN-α, MCP-1 and IL-8 in cell supernatants were analyzed by ELISA (R&D Systems or Amersham Biosciences) following the manufacturer's recommendations, and the phenotype of pDCs was analyzed by flow cytometry. Experiments carried out with cDCs were done in a similar way. In some experiments pDCs were cultured with supernatants harvested from confluent epithelial cell cultured during 12 h at 37°C. When indicated coculture of pDCs and epithelial cells were performed in 24-transwell chambers with a polycarbonate filter (0.2 µm pore size). In these experiments epithelial cells were grown to confluence on the filter, and pDCs (3×10^5^) were added in the upper chamber, enabling the contact between pDCs and epithelial cells, or in the lower chamber. After 12 h of culture, pDCs were harvested and their phenotype was analyzed by flow cytometry.

### Flow cytometry

Fluorescein isothiocyanate (FITC), phycoerythrin (PE), or allophycocyanin (APC) conjugated MAbs directed to CD1a, CD14, CD80, CD86, CD40, HLA-DR, CD83, CD123, TNF-α, IL-1β (BD Pharmingen, San Diego, CA), LL37 (Santa Cruz Biotechnology, Santa Cruz, CA), and TLR-9 (eBioscience, San Diego, CA). Rabbit polyclonal anti-TLR7 was from IMGENEX (San Diego, CA). In all cases, isotype-matched control antibodies were used, and a gate (R1) was defined in the analysis to exclude all nonviable cells and debris, based on size and propidium iodine staining. Analysis was performed by using a FACS flow cytometer and CellQuest software (BD Biosciences, San Jose, CA). The results are expressed as the mean fluorescence intensity or as the percentage of positive cells.

### Quantitation of cellular apoptosis by annexin-V binding and flow cytometry

It was performed using an apoptosis detection kit (Immunotech, Marseille, France). In brief, cells were labelled with annexin-V-FITC for 20 min at 4°C and with propidium iodide immediately before evaluation of fluorescence by flow cytometry.

### Transepithelial electrical resistance

To estimate the integrity of the HRTC monolayer, transepithelial electrical resistance (TEER) was measured as described [47], [48] using a Millicell-ERS electric resistance system (Millipore, Bedford, MA, USA). Briefly, HRTC were grown to confluence on a polycarbonate filter (0.2 µm pore size) in the upper chamber of a 24-transwell plate. Then, epithelial cells were incubated for 18 h together with pDCs (2×10^5^) in the upper chamber or in the absence of pDCs. TEER was measured at 0, 3 and 18 h after the addition of pDCs. Data were corrected for the resistance of the empty filter.

### HIV-1 infection assays

GHOST cells expressing CD4, CXCR4 and a Tat-dependent green fluorescent protein (GFP) reporter cassette were infected by HIV-1 IIIB (X4 tropic) while GHOST cells expressing CD4 and CCR5 were infected with HIV-1 BaL (R5 tropic). In all cases, cells were cultured with HIV-1 (50 ng p24) for 2 h at 37°C. Cells were then washed and pDCs (1×10^5^) (untreated or preincubated with HT-29 cells for 12 h) were added to GHOST cells. Infection of GHOST cells was analyzed by flow cytometry at 48 h post-infection by studying the percentage of GFP+ cells, as previously described [49]. To neutralize the activity of type I IFNs, a specific blocking antibody directed to the common receptor IFN-R was used (clone MMHAR-2, PBL interferon Source, NJ, USA). Infection of MT-2 cells was performed using HIV-1 IIIB and it was evaluated by measuring p24 antigen levels in cell supernatants at 48 h post-infection by ELISA.

### Statistical analysis

All statistical comparisons were performed by using one-way analysis of variance with Dunnett post-test. P values<0.05 were considered statistically significant.
